# Safety and Efficacy Endpoints for Mesenchymal Stromal Cell Therapy in Renal Transplant Recipients

**DOI:** 10.1155/2015/391797

**Published:** 2015-07-15

**Authors:** J. R. Bank, T. J. Rabelink, J. W. de Fijter, M. E. J. Reinders

**Affiliations:** ^1^Department of Nephrology, Leiden University Medical Center, Albinusdreef 2, 2333 ZA Leiden, Netherlands; ^2^Department of Einthoven Laboratory for Experimental Vascular Medicine, Leiden University Medical Center, Albinusdreef 2, 2333 ZA Leiden, Netherlands

## Abstract

Despite excellent short-term graft survival after renal transplantation, the long-term graft outcome remains compromised. It has become evident that a combination of sustained alloreactivity and calcineurin-inhibitor- (CNI-) related nephrotoxicity results in fibrosis and consequently dysfunction of the graft. New immunosuppressive regimens that can minimize or eliminate side effects, while maintaining efficacy, are required to improve long-term graft survival. In this perspective mesenchymal stromal cells (MSCs) are an interesting candidate, since MSCs have immunosuppressive and regenerative properties. The first clinical trials with MSCs in renal transplantation showed safety and feasibility and displayed promising results. Recently, the first phase II studies have been started. One of the most difficult and challenging aspects in those early phase trials is to define accurate endpoints that can measure safety and efficacy of MSC treatment. Since both graft losses and acute rejection rates declined, alternative surrogate markers such as renal function, histological findings, and immunological markers are used to measure efficacy and to provide mechanistic insight. In this review, we will discuss the current status of MSCs in renal transplantation with a focus on the endpoints used in the different experimental and clinical studies.

## 1. Introduction

Renal transplantation has improved life expectancy and quality of life for patients with end-stage renal disease. Improvements in surgical techniques and the introduction of novel immunosuppressive agents have improved the short-term outcome after transplantation markedly in the past decades [1]. Long-term graft survival, however, remains suboptimal, even in patients with low immunological risk for rejection [1, 2]. It has become evident that both immunological and nonimmunological factors adversely affect renal structure, including ischemia/reperfusion injury (IRI), subclinical rejections, viral nephropathy, and calcineurin inhibitor (CNI) overexposure [3], causing tubular atrophy and interstitial fibrosis (IF/TA). In addition, the currently used immunosuppressive drugs have several side effects including diabetes, hypertension, and nephrotoxicity and carry an increased risk for malignancies and (opportunistic) infections. Consequently, there is a strong interest in novel immunosuppressive therapies that have minimal side effects and prevent or reverse IF/TA in the allograft with the aim to prolong (allograft) survival.

A promising novel therapeutic option in this respect is the clinical application of mesenchymal stromal cells (MSCs). MSCs have important effects on the innate and adaptive immune system and possess anti-inflammatory properties [4]. In addition, MSCs can enhance repair by secreting antifibrotic and proangiogenic factors, which makes them attractive for potential use in renal transplantation. Animal studies in solid organ transplantation predominantly investigated cell-product efficacy and mechanisms of action. Several studies demonstrated a prolongation of allograft survival after MSC therapy [5–7] and an inhibition of the rejection process [8, 9]. In humans, rationale for use of MSCs in renal recipients includes a reduction in severity of IRI, prevention and reversal of acute transplant rejection, and reversal or stabilization of chronic transplant inflammation and fibrosis. In addition, adding MSCs to the immune suppressive regimen might facilitate CNI withdrawal with the aim of preserving renal function and structure [10, 11].

The early phase I clinical trials with MSCs focused primarily on safety and feasibility, with additional clinical parameters to get an impression on the biological effect of MSC therapy [12–17]. Phase II clinical trials, with a focus on efficacy of MSC treatment, have been started recently [11, 18]. One of the most difficult aspects is to define accurate endpoints, which can be considered to predict reliable the outcome after renal transplantation and which can measure efficacy of MSC treatment. The gold standard clinical endpoints in renal transplantation are patient and graft survival, biopsy proven acute rejections (BPAR), and renal allograft function [19]. However, substantial improvements in patient and kidney survival and declining acute rejection rates, have shifted the endpoints to alternative surrogate markers, such as histological findings and immunological markers [19, 20]. The most relevant endpoint markers might differ among clinical trials, depending on the rationale of using MSCs in renal transplantation. In this review, the current status of MSCs in renal transplantation is discussed with the focus on the chosen endpoints.

## 2. Preclinical Studies Of MSCs: Endpoints Mainly Focused on Graft Survival and Prevention of Rejection

MSCs are involved in a variety of physiological processes, including immune modulation and repair of injury [21, 22]. The immune modulatory potential of MSCs has most extensively been studied. MSCs inhibit T cell proliferation via several mechanisms including indoleamine 2,3-dioxygenase (IDO) activity, and the production of prostaglandin E2 and transforming growth factor- (TGF-) *β* [23–25]. Furthermore, MSCs alter cytokine secretion profiles of naïve and effector T cells [26–28]. Recently, it was shown that MSCs suppress not only the Th1 functions but also the Th17-mediated activation and proliferation through soluble and cell-dependent factors [29, 30]. Besides their effects on T cells, MSCs have additional targets in the immune system. They inhibit the interleukin- (IL-) 2 and IL-15 driven natural killer (NK) cell proliferation and interferon- (IFN-) *γ* production [31–34] as well as the dendritic cells (DC) generation from peripheral blood monocytes* in vitro* [28, 35, 36]. Interestingly, intravenous injection of MSCs significantly affected the ability of DCs to prime T cells* in vivo* because of their inability to home to draining lymph nodes [37]. In addition, MSCs have immune regulatory activities and are capable of inducing generation of CD4^+^CD25^+^FoxP3^+^ regulatory T cells via both cell contact dependent mechanisms and via the secretion of TGF-*β*1 and prostaglandin E2 [38].

Data on the role of MSCs in B cells are less extensively studied. MSCs have been shown to inhibit the differentiation of B cells; however, it remains elusive whether this is a direct or indirect effect [39]. There is also evidence that MSCs induce B cells with regulatory functions [40]. Recently, Franquesa et al. found that adipose tissue derived MSCs exert an indirect effect on B cell proliferation through immunomodulation of T cells and a direct effect on B cells by inhibiting plasmablast differentiation and induction of IL-10-producing regulatory B cells [41].

*In vivo*, several studies investigated the effects of MSCs in experimental models of solid organ transplantation. Most studies focused their endpoints on efficacy including prolonged graft survival [5–7] and inhibition of the rejection process [8, 9]. In addition to these endpoints, an important aim of the experimental studies was to elucidate mechanisms involved. Zhou et al. observed a prolonged heart allograft survival, which was associated with a suppressed allogeneic T cell response [6], where Casiraghi et al. found an association of prolonged cardiac graft survival in mice with the generation of regulatory T cells [7]. de Martino et al. investigated whether MSCs can downregulate the immune response and control the acute cellular rejection after rat kidney transplantation [8]. The MSC-treated rats had an improved kidney function and histologically tubular damage and vasculitis was diminished. In addition, MSCs reduced the number of ED1^+^ and CD8^+^ cells, showing that MSCs indeed can downregulate the immune response and attenuated histological damage.

Casiraghi et al. investigated in a murine kidney transplant model the best timing of autologous MSC infusion and explored the mechanism of the immune modulatory and/or inflammatory activities of MSC according to timing of MSC infusion [42]. They found that pretransplant MSC infusion significant prolonged kidney graft survival compared to posttransplant MSC infusion. In addition, the MSCs infused pretransplantation localized into lymphoid organs where they promoted early expansion of the regulatory T cells, in comparison to posttransplant infusion where MSCs localized in the graft. These results suggest that MSCs may modulate the immune response by shifting the balance between regulatory T cells and effector T cells to a more tolerogenic profile. This finding was confirmed in a study with kidney allograft mice by Ge et al., where CD4^+^CD25^+^Foxp3 were essential for tolerance induction [43]. In addition, the same group investigated the mechanisms for kidney allograft tolerance and found that the generation of regulatory T cells through IDO-expressing MSCs could be a potent mechanism involved.

Another important physiological function of MSCs is their role in angiogenesis and fibrosis. MSCs were shown to produce angiogenic factors that promote stabilization of the vessels, including vascular endothelial growth factor (VEGF) and angiopoietin-1 (Ang1) [44, 45]. In addition, MSCs can release hepatocyte growth factor and bone-morphogenic protein-7 (BMP-7), which are potent inhibitors of fibrosis [46]. Interestingly, in a rat kidney allograft model, Franquesa et al. observed a therapeutic effect of MSCs attenuating the progression of IF/TA when this process was already in progress [47]. Besides a reduction in IF/TA, MSC-treated animals demonstrated fewer macrophages infiltrating the parenchyma, lowered expression of inflammatory cytokines in combination with increased expression of anti-inflammatory factors.

The above described studies provide us important mechanistic information on MSCs in the transplant setting. Translation of the findings of experimental studies into the human situation is, however, difficult. The underlying disease of the renal recipient, the concomitant use of immunosuppressives, the difference in inflammatory responses between animals and humans [48], and numerous other factors which are of relevance for the human situation are not taken in account in the animal studies.

## 3. Safety Aspects Related to MSCs in Human Renal Transplantation

Safety is in general defined as a relative freedom from danger, risk, or harm. It should be clear that the safety of the patient is paramount. MSC isolation, expansion, harvesting, and cryopreservation should be performed under strict GMP conditions. In the different laboratories final cell products have to undergo control quality tests before release including viability, sterility, endotoxin content, mycoplasma contamination, fluorescence-activated cell sorting (FACS) analysis, and tests to ensure genetic stability. In addition, clinical studies should be performed under ethically approved protocols and appropriate Data Safety Monitoring Board oversight. Serious adverse events (SAEs) and suspected unexpected serious adverse reactions (SUSARs) should be carefully recorded and reported to the proper authorities. Here potential risks related to the MSC infusion, including direct toxicity of the MSC infusion, malignancies, risks for over immune suppression, and immunogenicity are discussed.

### 3.1. Potential Toxicity Related to the Infusion

To date, no direct toxicity related to the infusion itself or immediate adverse effects have been observed in the numerous clinical trials with MSCs for different clinical indications [49]. We are, however, still awaiting long-term effects. There is no uniform score yet, which can be used to assess safety in cell-based trials. In our phase I trial the WHO criteria were used to monitor toxicity and adverse events [14]. Of interest, Dillmann et al. proposed a scoring system to evaluate safety of intravenous and intraportal infusion of stem cell products after liver transplantations. The so-called MiSOT-1 score was designed to identify unacceptable treatment-emergent adverse events in phase I/II trials [50]. This score is developed to identify very serious adverse events; however, it is also of importance to identify the less severe adverse events, which are more likely to occur. For renal transplantation, no such score is developed yet.

### 3.2. Uncontrolled Proliferation

Renal transplant recipients have already an increased risk of malignancies due to the concomitant use of immunosuppressive medication [51]. The most common malignancies occurring in transplant recipients are skin cancers, especially squamous cell carcinomas [52]. The incidence of these carcinomas increases with the duration of immunosuppressive therapy and registry data shows that ultimately 50 percent of the white transplant recipients are affected [53]. This well-known risk of malignancies in combination with the proangiogenic, antiapoptotic, and immune modulatory properties of MSCs may act together as tumor promoters.* In vitro* and* in vivo* studies have shown that MSCs have a potential for mal differentiation into neoplastic cells as well as the possibility to promote growth of the tumor cells [54, 55]. MSCs were shown to migrate to the site of microscopic tumor lesions and to incorporate into tumor vessels [55]. By contrast, in several animal models with preestablished neoplastic disease, including non-Hodgkin's lymphoma models [56], gliomas [57], and Kaposi's sarcoma [58], the infusion of MSC exerted antineoplastic properties. However, since these are studies in immune compromised mice this may not simply be extrapolated to humans. In humans quite a large cohort of patients is exposed to MSC therapy and none of them developed new malignancies so far [59]. Even in patients where MSCs are used for hematological malignancies no tumors occurred [59]. However, most clinical trials have a short follow-up period and the inclusion of ill patients with poor prognosis could have biased the outcomes. We suggest that patients who have received MSC therapy should be monitored closely and there must be a low threshold for further research, for example, a CT or PET scan.

### 3.3. Opportunistic Infections

Opportunistic infections are another important aspect related to safety. In the study of Tan et al. a significant decrease in opportunistic infections was seen with MSC induction. However, 151 of the 154 patients in this study had a cytomegalovirus (CMV) negative serological status, probably explaining the low incidence of CMV infections in their population [18]. In contrast, in our safety and feasibility study a relatively high number of opportunistic infections were found, in 3 out 6 patients, which might be related to the immunosuppressive effects of MSCs [14]. Other clinical trials in the context of graft-versus-host disease (GVHD) or hematopoietic stem cell transplantation (HSCT) also showed a trend to more infections after MSC therapy. In a study by von Bahr et al. in 31 patients with GVHD and MSCs a high rate of infections was seen. However, most patients in this study were patients with steroid-refractory GVHD and, since there was no matching control group, infections could not be fairly compared [60]. In addition, MSC coinfusion after HSCT caused a higher 1-year incidence of infections, particularly fungal infections [61]. Thus frequent and accurate monitoring of infectious complications remains essential.

### 3.4. The Role of Allogeneic MSCs in the Transplant Setting

In humans, allogeneic MSCs have several advantages compared to autologous MSCs. Allogeneic MSCs are directly available which makes them applicable for patients who need a treatment without a delay. Another benefit of using allogeneic MSCs is that the product can be easily standardized and, therefore, provides more comparable results [62]. However, a potential danger of allogeneic MSCs and in particular donor-derived MSCs could be sensitization. Donor-MSCs can trigger an antidonor immune response against the graft and may lead to an increased risk for allograft rejection. In animal models, allogeneic MSCs had the capacity to induce antidonor immune response via indirect antigen presentation pathways and accelerated rejection of the graft [63]. Although the immunogenicity of allogeneic MSCs needs further study to prove safety in clinical trials, a recent clinical pilot study with donor-MSCs showed that it was safe to reduce the conventional Tacrolimus dose in living related kidney transplant recipients. Unfortunately, anti-human leukocyte antigen (HLA) specific antibodies were not obtained in this study [15].

Taken together, clinical trials with MSCs have to deal with a lot of safety aspects, given the significant challenges in processing MSCs. In addition, since transplant recipients have already an increased risk of (opportunistic) infections and malignancies due to the concomitant immunotherapy, it is very difficult to determine the additional risk of MSC infusions [51]. The key requirements in those early phase studies, therefore, include MSCs culturing with accurate manufacturing standards, sharing scientific expertise and early clinical data, monitoring study by an independent expert panel, and closely monitoring patient for a longer period of time.

## 4. Measuring Efficacy in Clinical Trials with MSCs

Since patient and kidney survival have markedly improved, and acute rejection rates have declined, these endpoints are nowadays difficult to use in the clinical setting. For example, to assess biopsy proven acute rejection (BPAR) rates as primary objective, sample size calculations indicate that at least 320 patients are necessary for a prospective randomized controlled trial to detect a reduction of 50% in rejection rate (assuming a rejection rate of 20% in the control group) with two-tailed significance of 0.05 and 80% power (chi-quadrate test). The production of MSCs is labor-intensive and costly, and such a design would be a great, if not almost impossible, challenge. Therefore, surrogate endpoint markers are used which can predict BPAR and graft survival, including histological findings, measurement of renal function, and immunological and cardiovascular markers depending on the design of the trial [19, 20]. According to the indication of MSC therapy the focus of the various endpoints might differ. For example, when assessing IRI, short-term endpoints such as renal function and BPAR will provide the most insight in efficacy. On the other hand, when assessing chronic allograft injury, the endpoints will be more focused on long-term outcomes and will include patient and graft survival, histology, and renal function. Other endpoints, including immune monitoring and biomarker studies, will apply for all studies, although the focus on the markers chosen may vary between trials. Therefore, it is of importance to establish the rationale first, and then the most relevant efficacy endpoints should be determined accordingly.

### 4.1. Histopathological Evaluation of Renal Biopsies after MSC Treatment

Since MSCs have anti-inflammatory and remodeling properties both* in vitro* and* in vivo* endpoints focusing on these processes are of paramount importance. Therefore, tissue analysis should include quantification of fibrosis and of inflammatory processes in the graft [2, 64, 65]. Pathologic evaluation of IF/TA is central in assessing the severity of chronic injury and it has been suggested that early histological detection of IF/TA may be a surrogate marker for the risk of graft failure [66]. In humans, a widely used histological scoring system is the Banff '07, which has grown to be the standard setting for pathologist to evaluate renal transplant biopsies. The Banff scoring system is updated on regular basis in response to emerging data and technologies and discussed by several pathologist, clinicians, and scientists. However, the Banff has also some limitations; the precise quantification of interstitial fibrosis is difficult with the Banff since the score is semiquantitative and studies showed that there might be a wide interobserver variability [67–69]. Another surrogate quantitative marker for the degree of fibrosis is computerized image analysis of fractional interstitial fibrosis of Sirius red stained biopsies. Sirius red staining is specific for collagen types I and III, which represent 80% and 15–20%, respectively, of the total collagen synthesized by fibroblasts [70]. Encarnacion et al., examined Sirius red stained tissue of 49 renal transplant recipients with established chronic allograft nephropathy and demonstrated that it significantly correlated with GFR measured by iothalamate clearance. Furthermore, several studies have indicated that Sirius red staining is an accurate and reproducible method to measure the degree of fibrosis [70, 72–74].

### 4.2. Renal Function

Renal function is used in the follow-up to detect graft dysfunction and to evaluate treatment. In most studies, renal function is estimated by serum creatinine levels. An analysis of more than 100.000 renal transplant recipients showed that creatinine values at 6 months and 1-year were correlated with long-term graft survival [75]. However, a major disadvantage of serum creatinine is that it is dependent on age, body weight, race, and sex. In absence of a renal biopsy, measured glomerular filtration rate (GFR) provides the most accurate analysis of renal function. In clinical practice, GFR can be estimated using different formulae, for example, MDRD (modification of diet in renal disease), CKD-EPI, and Cockcroft-Gault, or measured with 24-hour urine collection or radiological evaluation. The estimated GFR equations methods have been shown to improve the accuracy in the prediction renal function compared to serum creatinine alone. In a systematic review by Shaffi et al. it was shown that the CKD-EPI and MDRD equations are most accurate available equations in solid organ transplant recipients [76].

GFR can also be measured using 24-hour urinary creatinine values. Urine collection is a relatively precise method; however, it requires accurate collection of 24-hour urine for the patients. Another method to measure the GFR is by radiological evaluation with inulin, iothalamate, or iohexol. Those substances are freely filtered by the glomerulus and is neither secreted nor reabsorbed by the renal tubule. Of these, inulin clearance is the historical gold standard; however, this technique is intensive and its usefulness in clinical practice is limited. Iohexol, a nonradiolabeled contrast agent, is also currently used as a measure of GFR by calculating its plasma clearance after intravenous bolus injection. Iohexol clearance is a good alternative to inulin and showed a high degree of reproducibility over a wide range of renal function [77].

In renal transplant recipients, however, the performance of the GFR equation is suboptimal and it seems that tubular dysfunction contributes to this disagreement in measured and estimated GFR [78]. The greater the disturbed tubular function, the greater the difference between measured GFR and estimated GFR [79]. So the best method to assess renal function in renal transplant recipients remains by measuring GFR instead of estimating, especially in patients with tubular dysfunction. In trials with MSCs the determination of the renal function is of importance for both follow-up of renal function and assessing safety of the MSCs.

### 4.3. Biomarkers and Immune Monitoring Strategies

There is a critical need for biomarkers, which can early identify the diagnosis and the treatment response, and which can predict the outcome of (surrogate) endpoints in renal transplantation. A biomarker is defined as any objectively measurable parameter used to quantify a normal biological or pathological process. Intensive research has been done studying several biomarkers in kidney injury, including neutrophil gelatinase-associated lipocalin (NGAL), kidney injury molecule-1 (KIM-1), interleukin-18, and liver-type fatty acid-binding protein (L-FABP). Of these markers KIM-1 is of particular interest since KIM-1 is one of the best-characterized biomarkers in renal disease and transplantation [80, 81]. Moreover, KIM-1 is a marker of proximal tubular injury and it has been shown to promote apoptotic and necrotic cell clearance and to play an important role in renal recovery and tubular regeneration [82]. Upon ischemic injury, KIM-1 is upregulated and shed into the urine and extracellular space. Two to three days after injury a peak concentration is seen, congruent with the timing of repair [83, 84]. In the setting of MSC therapy, Franquesa et al. studied in a rat model the long-term beneficial effect of MSC injection in chronic allograft nephropathy and measured gene expression of KIM-1. A decreased expression of KIM-1 was found, indicating an injury blockage by the MSC therapy [47].

In addition to KIM-1, genomic and proteomic platforms have provided various promising new biomarkers during the last few years. A strong focus on development of biomarkers that can monitor safety, immune modulation, and regeneration should be the aim in MSC based trials. However, it is of importance to realize that there is no routine application of any of the biomarkers markers in clinical transplantation yet. In addition, the validation is still insufficient, probably due to the heterogeneity of the patients with kidney injury, the underlying etiologies and treatment strategies, and the patient's comorbidities. In addition, kidney injury is not a single disease entity but a multifactorial process [85]. Therefore, a single biomarker that reflects physiological and pathophysiological processes in the injured kidney has been proven to be a difficult quest.

Immune monitoring by flow cytometry is crucial in the evaluation of novel therapies in renal transplantation. Recently, the One study consortium has developed an immune monitoring strategy to compare the efficacy of different cell therapies, including procedures for whole blood leukocyte subset profiling by flow cytometry. This is a standardized method to monitor patients in clinical trials and facilitates fair and meaningful comparisons between trials, particularly trials of novel therapies, such as MSC therapy [86]. They developed 6 panels to analyze the immune response: panel 01 includes the general immune status; panel 02, the T cell subsets and the *αβ*+ T cells and *γδ*+ T cells; panel 03, the T cell activation; panel 04, the T cell memory and regulatory T cells; panel 05, the B cell subsets; and panel 06, the dendritic cell subsets. Using the standardized strategy of leukocyte profiling as proposed by the One study consortium to identify changes in leukocyte subsets will make it feasible to detect the effects of MSC therapies within and between multicenter trials and also between different clinical trials. In addition, functional assays, such as the mixed lymphocyte reaction (MLR) and measurements of different cytokines, should be performed to analyze donor-specific lymphocyte proliferation after MSC treatment.

### 4.4. Measuring Cardiovascular Endpoints

Cardiovascular disease is a major cause of morbidity and mortality after renal transplantation. Since the risk of graft failure especially declined, measuring cardiovascular mortality and morbidity is becoming increasingly relevant.

Compared to the general population, patients with chronic kidney disease have a higher cardiovascular risk [87]. Previous studies have shown that there is an independent and graded association between a reduced estimated GFR and the risk of cardiovascular events [88]. This increased cardiovascular risk is partly due to the high prevalence of traditional risk factors, such as hypertension and diabetes. The association of kidney function with cardiovascular risk is, however, also independent of these traditional cardiovascular risk factors [87].

In renal transplant recipients cardiac disease is the cause of death for 18–30% as shown in registry data [89, 90]. Renal transplant recipients have a high prevalence of traditional cardiovascular risk factors including diabetes, hypertension, dyslipidemia, and cardiovascular disease at the time of transplantation. Following transplantation immunosuppressive therapy such as steroids and calcineurin inhibitor therapy may further aggravate the existing risk factors or promote the development of new risk factors [91–93]. In addition, there are specific transplant-related risk factors such as acute rejection, delayed graft function, and poor kidney allograft function which further contribute to an increased risk for cardiovascular events [94–98].

Of interest, MSCs have also been used for several cardiovascular indications. As shown in various animal models with a myocardial infarction MSCs can reduce or reverse fibrosis and contribute to cardiac repair [99–105]. Also in heart allograft models, MSCs were beneficial and resulted in long-term allograft acceptance [106, 107]. Currently, in humans, many ongoing trials investigate MSCs for cardiovascular disease and therapeutic beneficial effects have been suggested [108–110]. In renal transplantation the administration of MSCs might also have an indirect effect on cardiac function by decreasing the side effects of the currently used immunosuppressive drugs and improving renal function.

## 5. Current Clinical Status of MSCs in Renal Transplantation

**Figure 1:**
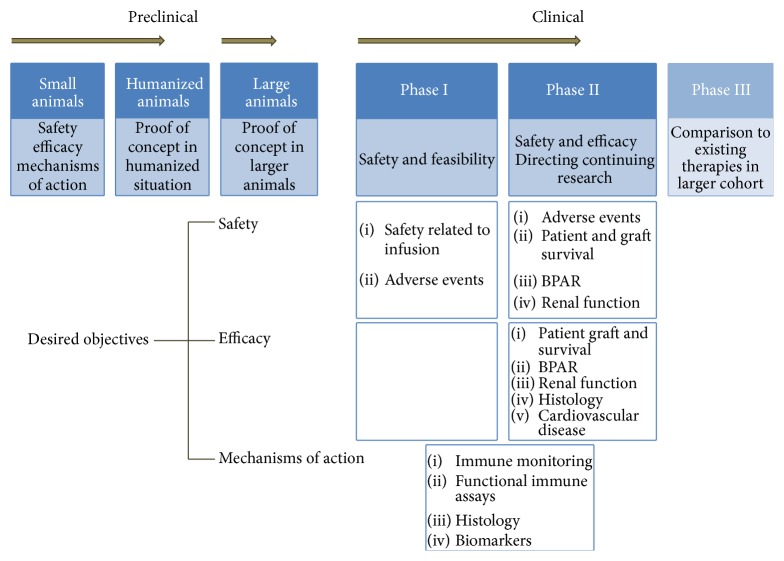
Desired objectives in clinical studies with MSCs in renal transplantation. Preclinical studies with MSC in the transplant setting start with small animals to investigate safety, efficacy, and mechanisms of actions. Then studies move on to prove the concept in humanized animals and larger animals. Human phase I studies address safety and feasibility in a low number of patients and determine the direction of further research. Phase II studies focus on both safety and efficacy parameters, which include patient and graft survival, BPAR, renal function, histology, and cardiovascular disease. Surrogate markers, such as immune monitoring and functional immune assays, are used to determine mechanisms of action. BPAR: biopsy proven acute rejection.

Numerous clinical trials with MSCs for various indications have been published so far, and different phases I and II trials are underway (Tables 1 and 2). Most clinical studies in renal recipients have focused so far primarily on safety and feasibility endpoints [12–17]. Safety in the different trials was defined as MSC infusion toxicity and/or adverse events related to MSCs with a follow-up period until 12 months after transplantation. Although the primary endpoints mainly focused on safety, the different studies have also assessed endpoints that provided insights into the mechanisms of actions of MSCs, as shown in Figure 1.

Different studies have focused on the role of autologous MSCs in the induction phase. MSC infusion was safe and clinically feasible [12, 13, 17, 18], although timing of the infusion seemed to be of major importance. In a pilot study by Perico et al. safety and clinical feasibility of autologous MSCs were tested in 2 kidney transplant recipients. MSC infusion was shown to be feasible, allowing enlarging of regulatory T cells in the peripheral blood, while restricting the memory CD8^+^ T cell expansion [12]. However, both patients given autologous MSCs after transplantation developed renal insufficiency, which was not observed when MSCs were administered before transplantation [13].

In our phase 1 clinical study, safety and feasibility of autologous MSC therapy was studied in patients with subclinical rejection or an increase in IF/TA in their renal biopsy at 24 weeks after renal transplantation (compared to the renal biopsy at 4 weeks). In total 6 of the 15 patients received MSC treatment, since not all patients met the inclusion criteria [14]. MSCs from patients with end-stage renal disease had similar phenotypical and functional characteristics compared to MSCs from healthy controls, as also reported for adipose tissue derived MSCs [111–113]. The MSC infusion was well tolerated and there were no adverse events related to the treatment itself. In addition, the initial results suggested immune suppression after MSC therapy. All patients that received MSCs demonstrated a profound reduction in proliferation of patient peripheral blood mononuclear cells (PBMC) 12 weeks after MSC infusion upon stimulation with donor specific PBMCs, while the response to third-party PBMCs was more variable. In addition, three patients developed opportunistic viral infections (2 CMV, 1 BK nephropathy), which might be related to the MSC treatment. In 2 patients with allograft rejection, there was a clinical indication to do a third biopsy. In both patients the infiltrate had disappeared and there were no signs of fibrosis after the MSC infusion [14].

In a recent clinical pilot study by Peng et al. allogeneic donor-derived MSCs were administered in 6 renal recipients for the first time. MSC infusion combined with low-dose Tacrolimus was safe and prevented acute rejection after renal transplantation [15]. Lee et al. also studied the safety and feasibility of allogeneic donor-MSCs injected directly into the bone marrow of the recipient's right iliac bone in 7 HLA-mismatched kidney transplant recipients. Two patients suffered from an acute cellular rejection, and one patient had an antibody-mediated rejection 9 days after transplantation [16]. In a recently registered safety study from our center renal transplant recipients will receive two doses of allogeneic third-party MSCs 6 months after transplantation (ClinicalTrials.gov identifier: NCT02387151). Primary objective is to evaluate whether allogeneic MSCs are safe by assessing BPAR and graft loss after MSC treatment. In addition, the development of de novo donor-specific antibodies (DSA) will be monitored. In this protocol specific criteria to minimize the risk of sensitization will be used, which includes no HLA sharing with the HLA mismatches of the kidney donor and no antibodies to the MSCs.

In a larger study by Tan et al., endpoints were primarily focused on efficacy. Living-related kidney transplant recipients (*n* = 159) were randomized to receive either MSC induction therapy with standard dose Tacrolimus, MSC induction with low-dose Tacrolimus, or interleukin-2 (IL-2) receptor blocker induction therapy with standard-dose Tacrolimus [18]. The primary outcome was the incidence of BPAR and renal function (MDRD) within the first year. BPAR with MSC induction was 8% compared to a relatively high acute rejection rate of 20% with IL-2 receptor blocker induction therapy. Although, with MSC induction, more late acute rejection episodes from 6 to 12 months were seen, up to 17%. There was no difference in BPAR between the low-dose and standard-dose Tacrolimus groups. In patients with MSCs induction a faster renal function recovery during the first month was seen compared to the standard Tacrolimus group. However, no improved renal function in the long-term was found. Secondary endpoints in this study included patient and graft survival and adverse events. Both patient and graft survival were comparable; however, the combined analysis of MSC-treated groups revealed significant decrease in opportunistic infections compared to the control group, as described previously.

In our study, which is currently running, autologous bone marrow MSCs will be used in combination with Everolimus with the aim of preserving renal structure and function in renal recipients. We hypothesize that the combination of MSCs with Everolimus might be an optimal strategy to facilitate early Tacrolimus withdrawal and reduce fibrosis compared to standard Tacrolimus dose [11]. Mammalian target of rapamycin (mTOR) inhibitors, such as Everolimus, have several benefits beside their immunosuppressive effects, which supports their clinical applicability. First, it has been shown that mTOR inhibitors reduce the incidence of CMV infections [114]. In addition, mTOR inhibitors exert antiproliferative effects and reduce the tumor burden [115–117]. In patients with only a single cutaneous squamous cell especially carcinoma conversion to mTOR inhibitor reduced the risk for development of future skin malignancies [116, 118, 119]. The primary endpoint in our study is to compare fibrosis by quantitative Sirius red scoring of MSC-treated and untreated groups at 6 months compared to 4 weeks after transplantation. Secondary endpoints focus on adverse events (including infections), BPAR and graft loss, renal function measured by iohexol, and progression of subclinical cardiovascular disease. In addition, immune monitoring will be performed according to the methods as standardized and validated in the One study [11, 86].

## 6. Summary

MSCs could potentially play an important role after renal transplantation in the prevention of acute rejection episodes, in the induction of tolerance, and in the prevention of IF/TA. Several animal models have investigated MSCs for those different indications and provided insights in the role and function of MSCs. In humans, the first phase I trials have been performed mainly with autologous bone marrow MSCs, demonstrating safety and feasibility. In addition, results indicated efficacy in preventing acute rejection, inducing long-term stable graft function and reducing tubulitis and IF/TA in small groups of patients. Currently, the first phase II trials with MSCs are recruiting patients, with an important focus on the minimization of immunosuppressive drugs in order to reduce fibrosis and to prolong allograft survival [10, 11]. In addition, studies with (matched) allogeneic MSCs are planned, which offer the advantage of availability for clinical use without the delay required for expansion.

One of the most important aspects in clinical trials is the definition of accurate endpoints. Patient safety is the cornerstone in each clinical trial. Strict follow-up of the possible risks of the therapy is needed. Although in current clinical trials no major side effects have been reported, longer follow-up of the MSC-treated patients is necessary in order to identify the possible long-term effects. To compare the effectiveness of MSCs well defined endpoints and appropriate controls are needed. Standardization on the different efficacy endpoints that are measured can cohere the different studies and facilitate fair and meaningful comparisons between trials. In this perspective, the standardized and validated methods for immune monitoring, as proposed by the One consortium, are a nice example. In general, sharing of procedures and protocols for safety and efficacy endpoints will allow for more reliable comparisons between the different clinical trials.

## Figures and Tables

**Table 1:** Objectives of currently registered and performed trials with autologous MSCs in renal transplantation.

Trial/study phase/outcome	Primary endpoint	Secondary endpoints
Induction therapy with autologous MSCs in living-related kidney transplants; phase II Lower incidence of BPAR, decreased risk of infections compared to IL-2RB induction [18].	(i) BPAR (ii) Renal function (MDRD)	(i) Patient and graft survival (ii) Adverse events

Autologous MSCs to induce tolerance in living-donor kidney transplant recipients; phase I Recruiting. ClinicalTrials.gov identifier: NCT02012153	Adverse events	(i) T cell counts (flow cytometry) (ii) Functional assays (ELISPOT in MLR) (iii) Regulatory T cell counts (flow cytometry) (iv) Urinary Foxp3 mRNA expression (qPCR)

Autologous MSCs under Basiliximab/low-dose RATG to induce renal transplant tolerance; phase I MSC administered at day 7 induced graft dysfunction. Not observed when MSCs were given day 1. Expansion of regulatory T cells and control of memory CD8+ T cell function [12, 13].	Safety related to MSC infusion	(i) Immunophenotyping T cells (Flow cytometry) (ii) Functional assays (ELISPOT for IFN-*γ* and Granzyme-B, cell-mediated lympholysis, HLA specific antibodies) (iii) Histology and Immunohistochemistry (graft infiltrating cells, MSC localization, complement deposition)

Autologous MSCs and subclinical rejection; phase I Feasible and safe. MSCs had immunosuppressive effects (3 patients suffered from opportunistic infections, 5 patients displayed downregulation of MLR), resolution of tubulitis [14].	(i) Adverse events (ii) Number of expanded MSCs (iii) Number of passages required	(i) Late acute rejections (ii) Histology (iii) Immunophenotyping T cells (flow cytometry) (iv) Functional assays (MLR, cytokines, HLA specific antibodies)

Autologous MSCs in combination with Everolimus to preserve renal structure and function in renal transplant recipients; phase II Recruiting [11]. ClinicalTrials.gov identifier: NCT02057965	Histology (fibrosis by Sirius red)	(i) Adverse events including (opportunistic) infections (ii) BPAR (iii) Graft and patient survival (iv) Renal function (iohexol, MDRD) (v) Immune monitoring (One study) (vi) Cardiovascular endpoints

Autologous MSC transplantation in the treatment of chronic allograft nephropathy; phases I-II Status unknown. ClinicalTrials.gov identifier: NCT00659620	Renal function (Cr and CrCl)	(i) Patient and graft survival (ii) Proportion of renal biopsies after 12 months (ii) Adverse events including (opportunistic) infections

Safety and efficacy of autologous MSCs transplantation in patients undergoing living-donor kidney transplantation; phase I Safe and feasible. Expansion of regulatory T cell population and reduced T cell proliferation [17].	Adverse events	(i) Immunophenotyping T cells (flow cytometry) (ii) Functional assays (proliferation assay) (iii) Renal function (Cr)

MSCs: mesenchymal stromal cells; BPAR: biopsy proven acute rejections; IL-2RB: interleukin-2 receptor blocker; RATG: rabbit antithymocyte globulin; Cr: creatinine; CrCl: creatinine clearance; MDRD: modification of diet in renal disease; HLA: human leukocyte antigen; MLR: mixed lymphocyte reaction; PCR: polymerase chain reaction; qPCR: quantitative PCR.

**Table 2:** Objectives of currently registered and performed trials with allogeneic MSCs in renal transplantation.

Trial/study phase/outcome	Primary endpoint	Secondary endpoints
Allogeneic MSC therapy in renal transplant recipients; pPhase I Recruiting. ClinicalTrials.gov identifier: NCT02387151	Safety (BPAR and graft loss)	(i) Histology (fibrosis by Sirius red) (ii) Renal function (iohexol, MDRD) (iii) (Serious) adverse events (iv) (Opportunistic) infections (v) Development of de novo DSAs (vi) Immune monitoring (One study)

Intraosseous injection of donor-derived MSCs into the bone marrow in living-donor kidney transplantation; a pilot study; phase I Injection into iliac bone was safe, no adverse events. Three patients suffered from acute rejection. Mixed chimerism was not detected [16].	Adverse events	(i) (Opportunistic) infections (ii) Histology (iii) BPAR (Banff) (iv) Chimerism analysis (STR-PCR) (v) Foxp3 quantitation (qPCR) (vi) Functional assays (MLR, cytokines)

Donor- derived MSCs with low-dose Tacrolimus prevents acute rejection after renal transplantation; phase I Injection of MSCs into renal artery was safe and feasible. Chimerism was not detected. More peripheral B cells in MSC group [15].	Safety of MSC infusion	(i) BPAR (ii) Graft function (Cr) (iii) Patient and graft survival (iv) Immune monitoring lymphocytes (flow cytometry) (v) Functional assays (MLR) (vi) Chimerism (STR-PCR)

Infusion of third-party MSCs after renal or liver transplantation; phases I-II Recruiting. ClinicalTrials.gov identifier: NCT01429038	(i) Safety of MSC infusion (ii) Incidence, timing, and severity of infections and malignancies	(i) Patient and graft survival (ii) Graft function (creatinine, HD after transplant) (iii) BPAR (iv) Immune function (flow cytometry, TREC quantification, V*β* repertoire diversity, and pathogen-specific T cells) (v) Anti-MSC and anti-organ donor HLA antibodies

MSCs: mesenchymal stromal cells; BPAR: biopsy proven acute rejections; Cr: creatinine; MDRD: modification of diet in renal disease; STR-PCR: short tandem repeat polymerase chain reaction; HD: hemodialysis; DSAs: donor specific antibodies; HLA: human leukocyte antigen; qPCR: quantitative PCR.
